# Feeding practices, nutritional status and associated factors of lactating women in Samre Woreda, South Eastern Zone of Tigray, Ethiopia

**DOI:** 10.1186/1475-2891-12-28

**Published:** 2013-03-01

**Authors:** Kiday Haileslassie, Afework Mulugeta, Meron Girma

**Affiliations:** 1 Department of Public Health, Mekelle University, P.O.Box: 1871, Mekelle, Ethiopia; 2 Department of Public Health, Mekelle University, Mekelle, Ethiopia; 3 Institute of Nutrition, Food Science and Technology, Hawassa University, Hawassa, Ethiopia

**Keywords:** South Eastern Tigray, Lactating mothers, Weighed food record, Feeding practices

## Abstract

**Background:**

Lactating mothers from low-income settings are considered as a nutritionally vulnerable group. Due to the nursing process, mothers are subjected to nutritional stresses. Frequent pregnancies followed by lactation increase the health risk of mothers resulting in a high maternal mortality.

**Objective:**

To assess the feeding practices, nutritional status and associated factors of lactating women from Samre Woreda, South Eastern Tigray, Ethiopia.

**Design:**

Community based cross-sectional survey

**Setting:**

Four kebeles of Samre Woreda (2 urban & 2 rural kebeles)

**Methods:**

Four hundred lactating mothers were recruited from 400 randomly selected households. Data on socio-demographic characteristics, maternal characteristics, feeding practices, frequency of foods eaten and dietary diversity was collected using a pre-tested and structured questionnaire. Anthropometric measurements were taken from each mother using calibrated equipments and standardized techniques. A one-day weighed food record was also collected from randomly selected sub sample (n=60) of mothers. The nutrient and energy content of foods consumed by the mothers was calculated by using ESHA Food Processor and the Ethiopian Food Composition Tables. To investigate the socio-economic and demographic factors affecting the nutritional status of the women, logistic regression was used. ANOVA and t-test were also used to see if there was a mean difference in nutritional status among the lactating mothers.

**Results:**

Majority (71.2%) of the participants did not take additional meals during lactation. The median dietary diversity score of the study participants was 5 out of 14 food groups. The prevalence of underweight, chronic energy deficiency and stunting were 31%, 25% and 2.2% respectively. Using logistic regression model, factors significantly associated with the nutritional status of the study participants (as determined by BMI and MUAC) were size of farm land, length of years of marriage, maize cultivation, frequency of antenatal care visit and age of breastfeeding child.

**Conclusions:**

The feeding practices, dietary intakes and nutritional status of the lactating women were short of the national and international recommendations. Therefore, sustained health and nutrition education is recommended to the women and their families and communities on increased food intake, proper dietary practices and dietary diversification during lactation in order to improve health and nutrition outcomes of lactating women.

## Introduction

Under nutrition and poor health from preventable causes disproportionately affect the well-being of millions of people in the developing world. Factors at individual, household and community level, or a combination of these factors, may contribute to poor nutrition and health status [1]. In particular, malnutrition among women is likely to have a major impact on their own health as well as their children’s health. More than 3.5 million women and children under age five in developing countries die each year due to the underlying cause of under nutrition [1].

Women are more likely to suffer from nutritional deficiency than men for several reasons, including their reproductive biology, low social status, poverty and lack of education. In addition, socio-cultural traditions and disparities in household work patterns can also increase women’s chance of being malnourished [2]. Between 5 to 20 percent of women in various African countries are underweight. Many African women display low weight-for-height as measured by a body mass index of less than 18.5 [3].

Women in low-income settings often consume inadequate amount of micronutrients because of resource limitation. They have a limited intake of animal source foods, fruits and vegetables. Intake of micronutrients less than the recommended values increase women’s risk of micronutrient deficiencies [4]. Adequate nutritional status of women is important for good health and increased work capacity of women themselves as well as for the health of their offspring [5].

Severely malnourished mothers have reduced lactation performance contributing to the increased risk of child mortality [6].

Nutritional requirements during lactation are greater than during pregnancy. If a mother is well nourished during pregnancy, she will have adequate fat and other nutrient reserves that can be used to compensate partially for her additional requirements. Mothers should be counseled about the need for an adequate diet in order to achieve optimal lactation and sustain it without depleting their own nutrient stores. Particular attention should be given to intakes of protein, calcium and vitamins [7].

Lactating mothers from low-income settings are considered as nutritionally vulnerable group. Due to the nursing process mothers are subjected to nutritional stresses. Frequent pregnancies followed by lactation increase the health risk of mothers resulting in a high maternal mortality [8]. A nursing mother produces 0.7 to 0.8 liters of milk per day, containing 330 milligrams of calcium per liter. This requires an extra energy expenditure of at least 500 calories per day [9]. The quality of breast milk is only affected in extreme cases of deprivation, or by excessive intake of a particular food [10]. But the quantity of milk depends very much on the mother's diet. Food absorbed by a nursing mother not only fulfills her own nutritional needs, which are greater during the postnatal period, but also enables her to produce milk [9].

Research based information regarding maternal nutrition from the study communities is lacking. Information on the feeding practices, nutritional status and associated factors of the lactating women are urgently needed for prioritizing, designing and initiating intervention programs aimed at improving maternal nutrition. The process for priority setting should start with the assessment and analysis of the situation that lactating women face in their environment. Thus, this study was carried out to provide information regarding the feeding practices, nutritional status and associated factors of the lactating women in the study area.

## Methods and materials

### Study area and population

The study was conducted in 2 rural and 2 urban kebeles (Kebele is the lowest administrative unit in Ethiopia consisting of about 1200 households) of Samre Woreda from March-April, 2011. The study area has a total population of 137,423 of which 70,086 (51%) were male, and 67,337 (49%) were female. Among these, 5222 (3.8%) were lactating mothers [11]. The study area is characterized by household food insecurity [11]. The total number of kebeles and schools were 23 and 48, respectively. The potential health service coverage in the study area was 85% [12]. The major agricultural products are wheat, barley, teff, sorghum, pea and beans and some vegetables like cabbage, carrots and lettuce [12].

### Study design and sampling

The study used a community-based cross-sectional survey design and data were collected from lactating mothers between the ages of 15-49 years. A sample size of 400 lactating mothers was calculated using a single population proportion formula with a 95% confidence level, 5% margin of error, 38% estimated prevalence of chronic energy deficiency in the study area [13], and by considering a non-response rate of 10%. The total kebeles in the woreda were initially stratified into rural and urban areas. Then, four (two from each strata) were selected from the existing 23 kebeles (4 urban & 19 rural) based on Probability Proportional to Size (PPS) sampling technique. The households in the selected kebeles with lactating mothers were identified through house-to-house visits by the research assistants. A sampling frame was prepared by registering all the identified eligible lactating women in each kebele. After that, simple random sampling was used to select the required number of lactating mothers.

### Data collection

#### Socio-demographic information

Trained female research assistants fluent in the local language (Tigrigna) administered the pre-tested questionnaire to selected lactating mothers in their own respective homes. The questionnaire was used to assess the socio-demographic characteristics, maternal characteristics, and the feeding patterns of the lactating mothers. Data on frequency of foods eaten and dietary diversity was also assessed using the questionnaire. Dietary diversity score (DDS) was collected and calculated as the sum of the number of different food groups consumed by the mother in the 24 hours prior to the assessment. A total of 14 food groups were considered in this study (i.e. cereals, vitamin A rich vegetables and tubers, white tubers and roots, dark green leafy vegetables, other vegetables, vitamin A rich fruits, any meat, egg, fish, legumes and nuts, milk and milk products, oils and fats, condiments and beverages, sweets) [14].

#### Dietary intake assessment

A one-day (24 hr) weighed food record was collected from randomly selected sub sample of study participants (n=60). The dietary assessment period lasted from the time the mother ate her breakfast to the time of dinner. For this reason, the trained female research assistants (four in each kebele) stayed in the study households from early morning until the mother’s last meal and weighed portions of the food consumed by the mother and any left over at the completion of each meal to deduct from the weight of the original serving. Foods and beverages consumed by the mother were weighed using digital scales (2 kg maximum weight: Model CS 2000, Ohaus Corporation, USA) accurate to±1 g. While weighing the foods and drinks, a detailed description of the foods and their cooking methods were recorded. To account for any day of the week effects on food and/or nutrient intake, weekends, weekdays and market days were proportionately represented in the survey. Study participants were well informed not to change their normal dietary pattern over the food record day. They were also asked to eat separately and not to share their meals during the day of data collection.

#### Anthropometric measurements

Weights of the lactating women were measured to the nearest 0.1 kg on a battery powered digital scale (Seca 770, Hanover Germany) and heights were measured to the nearest 0.1 cm using a wooden height-measuring board with a sliding head bar following standard anthropometric techniques [15]. Mid upper arm circumference (MUAC) was also measured using a non-stretchable MUAC tape [15]. For weight and height measurements, study subjects removed their shoes, removed their jackets and wore light clothing. Triplicate measurements of weight, height, and mid upper arm circumference were taken at the same day from each study subject using calibrated equipments and standardized techniques [15]. To avoid variability among the data collectors, all the anthropometric measurements were taken by the researcher (principal investigator). Body mass index (BMI) of the study subjects was calculated by dividing the weight in kilogram to the height in meter squared (kg/m^2^).

#### Data analysis

The nutrient and energy content of foods consumed by the mothers was calculated by using ESHA Food Processor and the Ethiopian Food Composition Tables [16,17]. Statistical analysis was carried out using SPSS for windows version 17.0 (SPSS Inc.). Continuous variables were checked for normality using the Kolmogorov-Smirnove test, scatter plots and histograms. Descriptive summaries using frequencies and proportions were used to present the study results. To investigate the socio-economic and demographic factors affecting the nutritional status of the women, logistic regression was used. Significant variables observed in the bivariate analysis were subsequently included in to the multivariate analysis. ANOVA and t-test were also used to see if there was a mean difference in nutritional status among the lactating mothers. P-values less than 0.05 were considered as significant.

#### Ethical considerations

The study protocol was reviewed and approved by the Institutional Review Board of the College of Health Sciences at Hawassa University. Permission to undertake the study was obtained from every relevant authority in the woreda. The nature of the study was fully explained to the study participants to obtain their oral informed consent prior to participation in the study and data was kept confidential. No resistance was made if a woman wanted to withdraw at any time from participating in the research.

#### Operational definitions

**Dietary Intake:** the amount of energy, nutrients or anti-nutrients available in the food consumed by the lactating mothers.

**Dietary Practice:** eating habit of the mothers during the time of their lactation.

**Lactating Mother:** mother who is currently feeding breast milk for her infant/child.

**Individual Diet Diversity Score**: is the sum of food groups eaten in a specified reference period, serves as a proxy of nutrient adequacy of an individual’s diet.

**Permanent Residents**: women who live in the study area for more than six months.

**Portion Size:** the amount of a food item consumed at a time.

**Women in the reproductive age:** relates to the standard recommendation of 15-49 years.

**Recommended Nutrient Intake:** also referred to as the Reference Daily Intake, is a term generally used to describe nutrient intake in regards to the majority of healthy people in a particular stage of life and of a particular gender.

## Results

### Socio-demographic characteristics of the study participants

A total of 400 lactating mothers aged 15-49 years were interviewed, with a response rate of 100%. The study participants included 200 lactating mothers from rural areas (kebeles) and 200 lactating mothers from urban areas. More than two thirds of the study participants, 275 (68.3%), were in the age range of less than or equal to 30 years and all of them (100%) belong to the Tigray ethnic group. Majority, 353 (88.25%) of the study participants lived in male-headed households. More than three fourths, 307 (77.3%) of the mothers in this study were housewives, whereas, only 93 (21.7%) were involved in income generating activities (Table 1).

**Table 1:** Socio-demographic characteristics of the study participants (n=400) in Samre Woreda from March-April, 2011

**Socio-demographic variables**		
Mean maternal age in years	28.6 (±6.5)^1^	
Age groups	n	%
17-25	149	37.5
26-35	198	49.9
36-49	53	12.6
**Maternal educational status**		
No formal education	243	60.8
Formal education	157	39.3
**Paternal educational status**		
No formal education	162	40.5
Formal education	238	59.5
**Maternal religion**		
Orthodox	369	92.3
Muslim	31	7.7
**Household head**		
Male	353	88.3
Female	47	11.8
**Family size**		
2-4 persons	146	36.5
≥ 5 persons	254	63.5
Mean	6	
**Maternal marital status**		
Married	369	92.3
Divorced	23	5.8
Widowed	8	2
Access to safe water (Public tap/protected spring)	354	88.6

^1^Mean ± SD (all such values).

### Maternal health and feeding practices

Majority of the study participants, (92%) received ante natal care (ANC) at least once during their last pregnancy, but only 45.5% had ANC visits of greater than or equal to 4 times (which is recommended). Slightly above half, (54.1%) of the study participants had 3-6 previous pregnancies. Almost all, 397 (99.3%) of the study subjects did not avoid any food because of cultural/traditional reasons, whereas, only 115 (28.8%) of them were eating additional foods during their lactation period (Table 2).

**Table 2:** Maternal health and feeding practices of the study participants (n=400) in Samre Woreda from March-April, 2011

**Maternal characteristics**	**n**	**%**
**Age of breast feeding child**		
< 6 months	150	37.5
6-11 months	90	22.5
12-24 months	142	35.4
> 24 months	18	4.4
**Number of gravidity**		
≤ 2 pregnancies	140	35
3-6 pregnancies	216	54.1
>7 pregnancies	44	10.9
**Practiced Exclusive Breast Feeding**		
Yes	319	79.8
No	81	20.2
**Last child’s sex**		
Male	202	50.5
Female	198	49.5
**Number of ANC attendance**		
No ANC visit	32	8
1-3 times	186	46.5
4-6 times	161	40.3
≥ 7 times	21	5.2
**Avoid eating any food during lactation**		
Yes	3	0.7
No	397	99.3
**Eat any additional foods during lactation**		
Yes	115	28.8
No	285	71.2
**Change in food intake during lactation**		
No change	285	71.2
Frequency of meal	32	8
Amount of meal	51	12.8
Both frequency and amount	32	8
**Number of meals/day**		
< 3 meals	108	27
≥ 3 meals	292	73

### Anthropometric status of the study participants

The mean height (±SD), weight (±SD), MUAC (±SD) and BMI (±SD) of the study participants were 156.0 cm (±5.4), 48.3 kg (±5.7), 23.2 cm (±1.9) and 19.8 kg/m^2^(±2.0), respectively. One hundred (25%) of the study participants had a BMI less than 18.5 kg/m^2^ (chronically energy deficient); whereas only 7 study participants had a BMI greater than or equal to 25 kg/m^2^ (over weight). Study subjects with a height of less than or equal to 145 cm (stunted) and with a weight of less than or equal to 45 kg (underweight) were 9 (2.2%) and 124 (31%), respectively (Table 3).

**Table 3:** Anthropometric status of the study participants (n=400) in Samre Woreda from March-April, 2011

**Anthropometric variables**	**n**	**%**
**Maternal height**		
≤ 145 cm	9	2.2
>145 cm	391	97.8
**Maternal weight**		
≤ 45 kg	124	31
>45 kg	276	69
**Maternal MUAC**		
< 21 cm	52	13
≥ 21 cm	348	87
Mean	23.2 (±1.9)^1^	
**Maternal BMI**		
< 18.5 kg/m^2^	100	25
18.5-24.99 kg/m^2^	293	73.2
≥ 25 kg/m^2^	7	1.8
Mean	19.8 (±2.00)^1^	

^1^Mean ± SD (all such values).

### Dietary intakes of the study participants

Majority of the study participants 369 (99.3%) consumed cereal based foods (made of teff, wheat, sorghum, millet, and barley). About three fourths 297 (74.3%) of the study participants reported that they consumed legumes prior to the survey and more than three fourths 366 (91.5%) of the study subjects consumed foods cooked with oil, fat or butter in the previous 24 hours. Other vegetables (tomato, onion) and dark green vegetables (kale and green pepper) were consumed by 347 (86.8%) and 158 (39.5%) of the study participants, respectively.

Other white vegetables and tubers (white potato, white sweet potato) and vitamin A rich vegetables (pumpkin, carrot) were consumed by 130 (32.5%) and 102 (25.5%) of the study subjects, respectively. Ninety four (23.5%) of the study participants reported that they consumed other fruits (banana, lemon and orange) where as fifty one (12.8%) reported that they consumed vitamin A rich fruits (ripe mango and papaya), a day prior to the survey. Among animal products, milk and milk products were consumed by 22 (5.5%) of the study subjects where as egg, flesh meat and organ meat were consumed by 20 (5%), 15 (3.8%) and 9 (2.3%), respectively.

### Comparison of the median energy and other selected nutrients intake of the women

Except iron (118 mg), which is by far higher than the recommended nutrient intake (10 mg), other median nutrient intakes as well as energy intakes of the lactating women in the study area were less than the recommended nutrient intakes of FAO/WHO/ UNU [18]. Vitamin C (23 mg) and vitamin A (194 micro grams) were considerably lower than the recommended nutrient intakes of the lactating women. The phytate content of the diet consumed by the lactating mothers in the study area was extremely high (1872 mg) (Table 4).

**Table 4:** **Median (25**^**th **^**and 75**^**th **^**percentile) energy and nutrient intakes of the lactating women in comparison with the EER and recommended nutrient intakes, (n=60)**

**Macro-and micro nutrients**	**Median (1**^**st **^**,3**^**rd **^**quartiles) intakes per day**	**Recommended dietary intake [**[18]**]**
Energy (Kcal)*	2031 (1689, 2279)	2365
Protein (g)	61 (51,73)	71
Calcium(mg)	662 (506, 831)	1000
Iron (mg)	118 (90, 298)	10
Zinc (mg)	9.2 (7.3, 10.3)	12
Thiamin (mg)	0.8 (0.6, 1.1)	1.5
Riboflavin(mg)	1 (0.8, 1.5)	1.6
Niacin (mg)	7.9 (5.7, 10.2)	17
Vitamin C (mg)	22.8 (14.7, 39.5)	95
Vitamin A ( RE-micro gram)	194 (101,326)	850
Phytate (mg)	1872 (1510, 2680)	__

* Energy requirement was estimated by assuming moderate physical activity.

### Socio-economic and demographic factors associated with the nutritional status (MUAC) of the lactating mothers

Size of farmland had a significant association on the nutritional status (MUAC) of the lactating mothers, i.e. those who had a land size of 0.26-0.75 hectare were 5.1 times more likely to be malnourished (MUAC < 21 cm) than those who had a land size of greater than 0.75 hectare [AOR=5.1,95% CI (1.06,24.46)]. Those whose years of marriage was between 11-20 years were 71% less likely to be malnourished (MUAC < 21 cm) than those whose years of marriage was less than or equal to 10 years [AOR=0.29, 95% CI (0.09, 0.9)]. Similarly, those who were not growing maize were 3 times more likely to be malnourished (MUAC < 21 cm) than those who were growing it [AOR=3, 95% CI (1.3, 7.2)].

But, nutritional status as determined by MUAC did not reveal any statistical association with family size, farm animal ownership, maternal educational status, number of meals per day, age of breast feeding child, number of parity and eating additional food during lactation (Table 5).

**Table 5:** Association of some socio-demographic variables with the nutritional status (MUAC) of the study participants (n=400) in Samre Woreda from March-April, 2011

	**Nutritional status (MUAC)**			
**Socio-demographic characteristics**	**Malnourished (< 21 cm)**	**Normal (≥ 21 cm)**	**Crude OR (CI)**	**Adjusted OR (CI)**
Land size	No (%)	No (%)		
≤ 0.25 hect.	9 (3.5)	83 (32.1)	1.9 (0.4,9.2)	1.5 (0.3,8.1)
0.26-0.75 hect.	24 (9.3)	106 (40.9)	3.9 (0.9,17.6)	5.1 (1.06,24.5)*
> 0.75 hect.	2 (0.8)	35 (13.5)	1	1
Family size				
2-4 persons	24 (6)	122 (30.5)	1	
≥ 5 persons	28 (7)	226 (56.5)	0.6 (0.4,1.1)	__
**Length of marriage**				
≤ 10 years	37 (9.3)	193 (48.7)	1	1
11-20 years	9 (2.3)	119 (30.1)	0.4 (0.2,0.8)*	0.29 (0.1,0.9)*
21-30 years	5 (1.3)	33 (8.3)	0.8 (0.3,2.2)	2.9 (0.7,11.9)
**Maternal education**				
Literate	22 (5.5)	135 (33.8)	1	
Illiterate	30 (7.5)	213 (53.3)	0.9 (0.5,1.6)	__
**Age of BF child**				
≤ 12 months	30 (7.5)	239 (59.8)	1	
> 12 months	22 (5.5)	109 (27.3)	1.6 (0.9,2.9)	__
**Growing maize**				
Yes	12 (4.7)	111 (43)	1	1
No	23 (8.9)	112 (43.4)	1.9 (0.9,4)	3 (1.3,7.2)*
**Number of gravidity**				
< 4 pregnancies	33 (8.3)	176 (44)	1	
≥ 4 pregnancies	19 (4.8)	172 (43)	0.6 (0.3,1.1)	__
**Own farm animals**				
Yes	26 (6.5)	209 (52.3)	1	
No	26 (6.5)	139 (34.8)	1.5 (0.84,2.7)	__
**Number of meals/d**				
< 3 meals	14 (3.5)	94 (23.5)	0.9 (0.5,1.9)	__
≥ 3 meals	38 (9.5)	254 (63.5)	1	

*P-value is significant at < 0.05.

### Socio-economic and demographic factors associated with the nutritional status (BMI) of the lactating mothers

Frequency of ANC visit had a significant association with the nutritional status (BMI) of the lactating mothers, i.e., women who had ANC visits of less than or equal to 3 times were 2.9 times more likely to be malnourished (BMI < 18.5 kg/m^2^) than those who had more than 3 ANC visits [AOR=2.9, (1.2, 7)]. Similarly, women who had children aged greater than 12 months were 2.8 times more likely to be malnourished (BMI < 18.5 kg/m^2^) than those who had children aged less than or equal to 12 months [AOR=2.8, (1.4, 5.8)].

But, nutritional status of study participants according to BMI classification had no significant association with maternal educational status, household asset (radio) ownership, family size, maternal age, number of meals per day, number of parity, farm animal ownership and residence (Table 6).

**Table 6:** Association of some socio-demographic variables with the nutritional status (BMI) of the study participants (n=400) in Samre Woreda from March-April, 2011

	**Nutritional status (BMI)**			
**Socio-demographic characteristics**	**Malnourished (<18.5 kg/m**^**2**^**)**	**Normal (≥18.5 kg/m**^**2**^**)**	**Crude OR (CI)**	**Adjusted OR (CI)**
Educational status	No (%)	No (%)		
Literate	44 (11.2)	109 (27.7)	1	
Illiterate	56 (14.2)	184 (46.8)	0.8 (0.5,1.2)	__
**ANC visits**				
≤ 3 times	45 (12.5)	139 (38.5)	1 (0.6,1.6)	2.9 (1.2,7)*
> 3 times	44 (12.2)	133 (36.8)	1	1
**Family size**				
2-4 persons	43 (10.9)	100 (25.4)	1	
≥ 5 persons	57 (14.5)	193 (49.1)	0.7 (0.4,1.1)	__
**Owner of radio**				
Yes	64 (16.3)	165 (42)	1	
No	36 (9.2)	128 (32.6)	0.7 (0.5,1.2)	__
**Age of BF child**				
≤ 12 months	50 (12.7)	215 (54.7)	1	1
> 12 months	50 (12.7)	78 (19.8)	2.8 (1.7,4.4)**	2.8 (1.4,5.8)**
**Maternal age**				
17-30	69 (17.6)	201 (51.1)	1	__
31-45	31 (7.9)	92 (23.4)	0.98 (0.6,1.6)	
**Number of gravidity**				
< 4 pregnancies	56 (14.2)	148 (37.7)	1	
≥ 4 pregnancies	44 (11.2)	145 (36.9)	0.8 (0.5,1.3)	__
**Residence**				
Urban	48 (12.2)	146 (37.2)	1	
Rural	52 (13.2)	147 (37.4)	1.1 (0.7,1.7)	__
**Number of meals/d**				
< 3 meals	26 (6.6)	80 (20.4)	0.9 (0.6,1.6)	
≥ 3 meals	74 (18.8)	213 (54.2)	1	__

*P-value is significant at < 0.05.

**P-value is significant at < 0.01.

## Discussion

This study assessed the feeding practices, nutritional status and associated factors among lactating women from Samre woreda, South Eastern Zone of Tigray, Ethiopia. According to the essential nutrition action (ENA), taking at least two additional meals per day during lactation is recommended for all lactating women [19]. Nevertheless, slightly below three fourth’s 285 (71.3%) of the study participants do not take any additional meal during their lactation time. The median dietary diversity score of the study participants was 5.0, which was lower than half of the food groups on FAO food grouping i.e. 14 food groups [14].

The prevalence of chronic energy deficiency (BMI <18.5 Kg/m^2^) among the lactating mothers was 25%, a prevalence which was very much lower than that of the Tigray region finding in the EDHS report of 2005 (37.5%) [13]. The probable reason for this discrepancy could be the interventions on maternal health, nutrition and other women empowering programs by the government as well as other non-governmental organizations in the study area.

But, this finding was comparable with the finding of the Ethiopian Health and Nutrition Research Institute nutrition baseline survey report (28.8%) [20]. On the contrary, our results were much lower than that of the prevalence in lactating mothers from the Southern region of Ethiopia (37%) [21]. The proportion of lactating mothers with a MUAC of less than 21 cm was 13%. This figure was lower than the finding of the survey report on the situation analysis of women in Tigray in 2011 (18.1%) [22].

The dietary assessment from one day weighed food record showed that the energy and almost all nutrient intakes of the lactating women in the study area were below the recommended nutrient intakes of FAO/WHO/UNU [18]. This finding was consistent with the finding of Huffman and his colleagues [23]. According to this study, many women in Africa suffered from chronic energy deficiency, inadequate weight gain during pregnancy and poor nutritional status. The additional energy needed for lactation is 20-25% of energy needs in the non-pregnant non-lactating state [24]. But, in the current study these energy needs were not met or were below the requirement of the lactating mothers. This might be due to their involvement in energy demanding workloads during the whole course of lactation and taking no additional meals from available foods in the household during this time.

The protein intake of the study participants was close to the recommended intake of FAO/WHO/UNU [18]. The quality of the dietary protein can be improved by combining protein sources (grains and legumes) with different limiting amino acids [25]. Thus, mixtures of plant proteins can serve as complete and well balanced source of amino acids for the lactating women from the study communities. In agreement with our findings, inadequate protein intake in lactating women was reported from Southern region of Ethiopia [21] and Malawi [26], where lactating women were predicted to be at risk of inadequate intakes of dietary protein.

The current study also revealed that micronutrient intakes especially zinc and calcium of the lactating women were below the recommended levels. However, iron intake (118 mg) was above the recommended intake of the FAO/WHO/UNU [18]. This was comparable to a study done in Tigray by Adish and his colleagues [27]. This may be due to the high content of iron in the Ethiopian diets as has been emphasized by Gebremedhin and his colleagues [28]. According to this study, the consumption of the indigenous cereal teff (*E.teff)*, oleaginous seeds and Ethiopian kale (*B. carinatabraun*), and all foods that appear to contain relatively high levels of iron enhance increased iron intake.

In the present study, it was also found that dietary zinc intake of the lactating women was lower than the recommended intakes of FAO/WHO/UNU [18]. This might also be related to the much lower intake of animal source foods that contain highly bio-available zinc than the foods of plant origin. Yewelsew and her colleagues also reported low dietary zinc intake in pregnant women from Sidama Zone, Southern Ethiopia [29]. As it was observed with the other micronutrients, the calcium intake was also lower than the recommended nutrient intake in the study participants. Inadequate intake of calcium among pregnant women from Sidama Zone, Southern Ethiopia was also reported by Yewelsew and her colleagues [29]. The majority of the study subjects were consuming cereal based foods (99.2%) and legumes (74.2%), which are known to contain significant amount of phytate that reduces the bioavailability of the zinc, iron and calcium absorption [30].

Women of reproductive age are thought to be vulnerable to vitamin A deficiency during pregnancy and lactation [31,32]. In the current study, it was found that the lactating women’s dietary intake of vitamin A (194 micro grams) was very much lower than the recommended intake (850 micro grams). The much lower intake of animal source foods (for vitamin A) as well as vitamins A and C rich fruits and vegetables might explain the lower intake of vitamin A in the lactating women.

Socio-economic and demographic factors affecting the nutritional status of the participants were size of farm land, length of years of marriage, maize cultivation, frequency of ANC visit and age of breastfeeding child. Those who had a land size of 0.26-0.75 hectares were 5.1 times more likely to be malnourished (MUAC < 21 cm) than those who had a land size of greater than 0.75 hectares. The strong association between land size and nutritional status might be explained by the fact that those who had a larger land size had an increased and diversified crop production as compared to those who had a smaller land size. Similarly, those whose years of marriage was between 11-20 years were 71% less likely to be malnourished (MUAC < 21 cm) than those whose years of marriage was less than or equal to 10 years. By the same token, the association between length of years of marriage and nutritional status could be explained by early marriage, i.e. those who were in a marriage for less than or equal to 10 years were mostly adolescents. A study by Teller and Gugsa conducted in Ethiopia reported similar finding, i.e., adolescent women were more likely to be malnourished compared to the other groups [33]. Furthermore, growing of maize was also associated with the nutritional status (using MUAC) of the study participants, i.e., those who were not growing maize were 3 times more likely to be malnourished (MUAC < 21 cm) as compared to those who were growing maize. The probable reason could be because of the diversified cultivation of crops as those who were growing maize had better land size than those who were not growing maize.

Study conducted in Hadiya Zone showed that religion, ethnicity, household size, age and parity, land and livestock holding were not associated with malnutrition, as measured by BMI and MUAC. Instead, level of education, sickness and estimated production of staple crops were found to be significantly associated with malnutrition [6]. In this study, nutritional status classified as MAUC < 21 cm or ≥ 21 cm did not reveal any statistical association with regard to family size, farm animal ownership, maternal educational status, number of meals per day, length of breast feeding, number of parity and eating additional food during lactation.

On the other hand, women who had ANC visit of less than or equal to 3 times were 2.9 times more likely to be malnourished (BMI < 18.5 kg/m^2^) than those who had more than 3 ANC visits per pregnancy. This might be because those who frequently visit the health institutions were getting health and nutrition educations as well as advice by the respective health professionals. Similarly, those women who had children aged greater than 12 months were 2.8 times more likely to be malnourished (BMI < 18.5 kg/m^2^) than those who had children aged less than or equal to 12 months. This could also be because of the increased nutritional requirement of the growing child but not increased food intake by the mother. Other probable reasons could also be giving less attention/care to the mother, work load and closely spaced pregnancies.

But, nutritional status of study participants according to BMI classification had no significant association on maternal educational status, household asset (radio) ownership, family size, maternal age, number of meals per day, number of parity, farm animal ownership and residence of the lactating mothers. This was in contrary to the study conducted on women’s nutritional status which had significant association on marital status, household assets, age of women and maternal education [33].

Major strengths of this study were the community based approach and random selection of the study households. This may made generalization possible to the study communities as an attempt was made to identify randomized households and lactating women from the study communities. Though weighed food record method is considered as a “gold” standard method for dietary assessment, the presence of data collectors in women’s homes for collecting weighed food records might alter the feeding behavior of the mothers, which may be considered as one of the limitations of this study. The seasonal variation of food availability that will have an effect on dietary intake and diversity was not considered due to the cross sectional nature of the study. Furthermore, it was difficult to establish a cause-effect relationship between the dependent variable (nutritional status) and the independent variables though association was observed.

## Conclusions

Majority of the lactating women (71.2%) who participated in the current study reported that they were not taking additional meal during the time of lactation. This study revealed that the energy and other nutrient intakes of the lactating women in Samre Woreda were below the recommended nutrient intakes of FAO/WHO/UNU [18]. Based on the anthropometric assessment, the prevalence of chronic energy deficiency (BMI <18.5 Kg/m^2^) among the lactating mothers was 25%, which is a high prevalence according to the WHO Expert Committee of 1995 [34]. The proportion of women whose height is less than or equal to 145 cm and whose weight is less than or equal to 45 kg was 2.2% and 31%, respectively.

Dietary intakes which were below the recommendation might lead the study subjects to poor nutritional status. Furthermore, the situation can be aggravated by the high phytate content food consumption of the study participants such as cereal based foods and legumes reported in the 24 hr recall and observed from the weighed food record. The important risk factors/predictors of having a MUAC <21 cm (under nutrition) were size of farm land, length of years of marriage and growing of maize. On the other hand, frequency of antenatal care and age of breast feeding child were the important predictors/risk factors for having a BMI of < 18.5 kg/m^2^.

In general, the feeding practices, dietary intakes and nutritional status of the lactating women in the study area were short of the national and international recommendations and were not adequate to support their increased energy and nutrient requirements. This implies the need for a sustained health and nutrition education to the women, their families and communities regarding increased food intake, proper dietary practices and dietary diversification during their lactation time in order to improve their health and nutrition outcomes. Thus, efforts should be made to improve the ANC services, diversify diet, increase consumption of vitamin A and C rich fruits and vegetables and reinforce traditional household technologies such as cooking, soaking, fermenting and sprouting to reduce the phytate content of the cereals and legumes consumed by majority of the study participants. Furthermore, research is required to determine the factors impeding the transfer of health and nutrition education in to action as well as to assess the dietary adequacy of the lactating mothers in the study area.

## Competing interests

We, the authors, have no any competing interests.

## Authors’ contribution

KH: initiation of the study, design, implementation, analysis and writing. AM: design, implementation, analysis and writing. MG: design, implementation and co-writing. All authors read and approved the final manuscript.
